# Patient safety measurement tools used in nursing homes: a systematic literature review

**DOI:** 10.1186/s12913-022-08814-5

**Published:** 2022-11-19

**Authors:** Kyoung-A Kim, Jungeun Lee, Dahee Kim, Deulle Min

**Affiliations:** 1 Department of Nursing, College of Nursing, Gachon University, 21936 Incheon, Republic of Korea; 2 College of Nursing, Cheju Halla University, Jeju, Republic of Korea; 3 The Graduate School, Wonkwang University, Iksan, Republic of Korea; 4 Department of Nursing, College of Medicine, Wonkwang University, 460, Iksandae-ro, 54538 Iksan, Jeonbuk Republic of Korea

**Keywords:** Patient safety culture, Older adults, Nursing homes, Tools

## Abstract

**Background:**

An increase in the number of older adults has highlighted the important issue of the safety of residents in nursing homes. This review aimed to review previous studies on patient safety of older adults living in nursing homes, analyze the tools used to measure it, and identify factors affecting patient safety of older adult residents in nursing homes.

**Methods:**

A literature search was conducted using EMBASE, PubMed, CINHAL, and COCHRANE. The main search terms were “nursing home” or “skilled nursing facility” or “long-term care facility” and “patient safety.” In total, 13,586 articles were identified. Two authors independently assessed the quality of each selected study using the Crowe Critical Appraisal Tool.

**Results:**

Twenty-five studies were included in the analysis. There were a total of seven tools used to measure patient safety in nursing homes: the Nursing Home Survey on Patient Safety Culture (10 studies) and Hospital Survey on Patient Safety Culture (nine studies). Furthermore, the Nursing Home Survey on Patient Safety Culture-China, Safety Attitudes Questionnaire, Safety Attitudes Questionnaire in a Skilled Nursing Facility, Safety Attitudes Questionnaire-Ambulatory Version, and Modified Stanford Patient Safety Culture Survey Instrument were used in one study each. The most used tool among them was the Nursing Home Survey on Patient Safety Culture. Most tools used to measure patient safety in nursing homes were related to patient safety culture and employee attitudes.

**Conclusion:**

Organizational factors, such as the staff education system and the composition of appropriate personnel, should be strengthened to establish a patient safety culture in nursing homes, for which policy support is crucial.

## Background

In 2019, 703 million people (about 9% of the world’s population) were aged 65 or over worldwide; this number is projected to increase to 1.5 billion (about 16%) by 2050 [1], suggesting that, by 2050, one in six people worldwide will be an older adult. Simultaneously, the number of older adults with chronic diseases has also increased, with 31.7% of the 9,432 older adults in China in 2015 having had one or more chronic diseases [2]. US studies have found that older adults with major chronic conditions—such as cardiovascular disease, cancer, and chronic respiratory disease—have a higher incidence of disability in activities of daily living [3]. This increase in the aging population has added to the burden on the social welfare system, with the US spending an additional $135.7 billion from 1996 to 2013 [4]. Recently, US Medicare and Medicaid Services reported that due to an aging population, the proportion of national health expenditures exceeded 15% of gross domestic product (GDP) in 2016 and will reach 19.4% (approximately $6 trillion) of GDP by 2027 [5].

Nursing homes are care facilities where older adults with physical or cognitive disabilities live while receiving professional support until death, with approximately 70% of people with dementia in the US receiving care during their final stages of life in such facilities [6, 7]. To improve and maintain the quality of care in these nursing homes, many countries around the world have devised quality indicators and implemented institutional evaluations, often fusing the concepts of quality of care and patient safety [8]. Most nursing home quality indicators include physical and mental safety indicators for residents, such as falls, severe pain, bedsores, urinary tract infections, physical restraints, premature death, emergency room presentations, delirium/dementia, weight loss/malnutrition, and drug-related events [8–10].

Several studies have reported that organizational culture emphasizing the importance of quality improvement and patient safety is an important factor that influences the care quality of nursing homes [11, 12]. An analysis of the relationship between patient safety culture (PSC) and nursing home ratings in 186 nursing homes across the US in 2016 reported that PSC significantly affected healthcare quality [11]. Another survey of 1,447 facility managers working in 818 nursing homes found that higher PSC resulted in fewer customer complaints and lower fines [12].

However, some studies have reported that organizational culture or climate for patient safety did not actually improve residents’ quality indicators [13, 14]. To the best of our knowledge, there has been no consensus on the factors affecting patient safety among nursing home residents. Another peculiarity is that different tools are used to measure the same patient-safety-related content. In a study that measured PSC in 2017, the Nursing Home Survey on Patient Safety Culture (NHSPSC) tool was employed [15], but in a Norwegian study in 2016, the Safety Attitudes Questionnaire (SAQ) tool was employed [16]. Therefore, the purpose of this study is to (1) review previous studies on patient safety of older adults living in nursing homes, (2) analyze the tools used to measure patient safety, and (3) identify the factors affecting patient safety of older adult residents in nursing homes. The results of the study will contribute to devising strategies to improve their quality of life.

## Methods

### Search strategy

The review process was made in line with PRISMA guidelines [17]. A literature search was conducted using the following databases: EMBASE, PubMed, CIHNAL, and COCHRANE. The main search terms were “nursing home” or “skilled nursing facility” or “long-term care facility” and “safety” or “patient safety.” Articles published at any time and in any country were considered. To develop a comprehensive search strategy, an effort was made to ensure that there were no documents that could potentially be missed in the database search. This was ensured by performing a search using terms from Medical Subject Headings or keywords mentioned in the references related to patient safety.

### Inclusion and exclusion criteria

The following criteria for inclusion in the literature review were used: nursing home participants, topics related to patient safety or safety, focusing on primary research, and English publications. Exclusion criteria were short-term residential care homes, visit home care, gray literature, instrument development, scoping review, and literature that did not use instruments to measure patient safety in nursing homes.

### Study selection

We handled literature using a literature management program EndNote 20 version (The EndNote Team, 2013, Philadelphia, PA, Clarivate) [18]. After discarding duplicate articles using the software, two researchers (JL and DK) independently conducted the selection and exclusion processes. The two researchers conducted all titles and abstract sifting for half of the papers and continued to share opinions. Disagreements between the researchers were resolved through discussion. The discussion continued until an agreement was reached. In addition, by placing different researchers in charge at each stage, we checked each other’s results.

### Data extraction and analysis

Two authors (DM and KK) validated the extracted data and resolved any disagreements. Previous literature on data selection was referred to in a systematic literature review [19], and a structured format was developed to ensure uniformity of the extracted data. Data on the following were extracted using data-charting forms: first author’s last name, publication year, country, sample size, study design, population, tool of measurement, quality assessment scores, study aim, and main result. The extracted data were then synthesized to summarize and investigate the current status of tool use, related factors, and implications for patient safety in nursing homes. The synthesized data are presented in tables describing the characteristics of the selected studies and their outcomes.

In addition, this study analyzed other tools based on the domain of HSOPSC version 1.0. HSOPSC is a reliable and valid tool developed by Agency for Healthcare Research and Quality (AHRQ). The tool was modified to version 2.0 in 2019 after releasing version 1.0 in 2004 [20], but version 1.0 is still used in many studies [21, 22]. It consists of the following 12 domains; Communication openness; Feedback and communication about error; Teamwork within units; Non-punitive response to error; Organizational learning; Supervisor/manager expectations and actions promoting patient safety; Staffing; Teamwork across units; Handoffs and transitions; Management support for patient safety; Frequency of events reported; Overall perceptions of safety [23].

### Quality assessment

Two authors (KK and DM) assessed the quality of each selected study using the Crowe Critical Appraisal Tool (CCAT) version 1.4 [24]. The CCAT is a validated instrument that has been widely used in systematic reviews. The study design that is used does not affect the assessment. All categories had to be scored; the lowest score for a category is 0 and the highest score is 5. At first, the agreement between authors for 25 papers was 88%. Any potential discrepancies that may arise during this process were resolved through discussion among the authors.

## Results

**Fig. 1:**
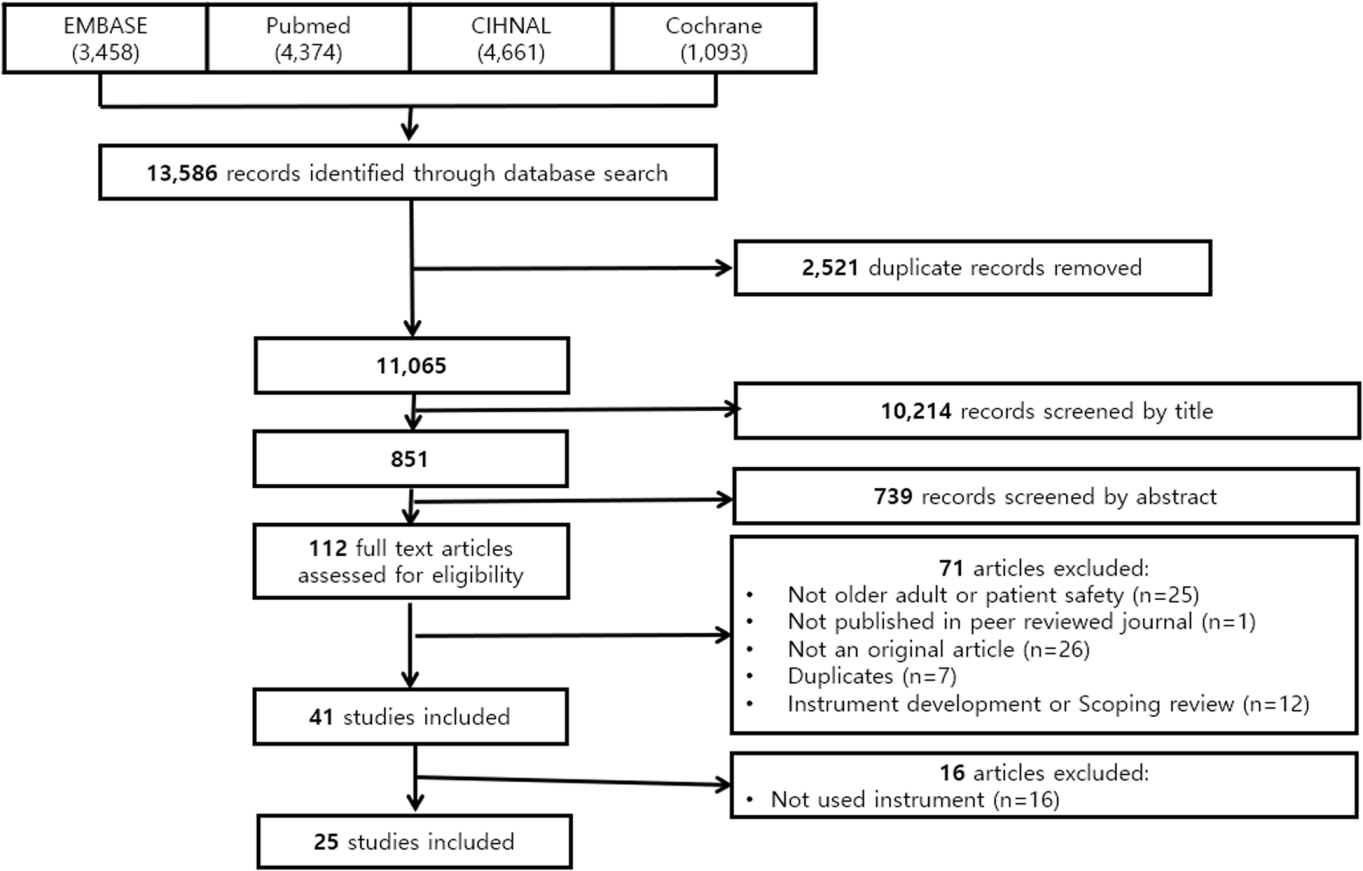
Process of literature selection

A total of 13,586 articles were identified in the primary search: EMBASE returned 3,458 articles; PUBMED 4,374; CIHNAL 4,661; and COCHRANE 1,093. After discarding 2,521 duplicate papers, we performed a selection and exclusion process for 11,065 papers. In the first and second selection and exclusion rounds, 10,214 articles were excluded after reading their titles and, following the third and fourth rounds, a further 739 articles were excluded after reviewing the abstracts. After reviewing the original text and excluding 71 papers that did not meet the selection criteria of this study, 41 papers remained. Among them, 25 papers were included in the final analysis, excluding 16 that did not include the use of patient safety tools (Fig. 1).

### Characteristics of the selected studies

Table. 1 presents the detailed characteristics of the 25 studies included in this systematic review. The NHSPSC and the Hospital Survey on Patient Safety Culture (HSOPSC) were used in 10 [11–13, 15, 25–30] and nine studies [21, 22, 31–37], respectively. In addition, the NHSPSC-China, Safety Attitudes Questionnaire (SAQ), Safety Attitudes Questionnaire in a Skilled Nursing Facility (SAQ-SNF), Safety Attitudes Questionnaire–Ambulatory Version (SAQ-AV), and Modified Stanford Patient Safety Culture Survey Instrument (MSI) were used in one study each. Of the 25 studies, 16 were conducted in the US [11–13, 15, 21, 22, 25, 27–29, 33–38], five in Norway [16, 30, 31, 39, 40] and the rest in France [26], China [32], the Netherlands [41], and Canada [42]. Most were quantitative studies, and there was one mixed study [42]. With regard to the participants, one study included long-stay nursing home residents with dementia [29], five studies targeted or included administrators [12, 15, 27, 34, 36], and the rest were conducted among facility staff working at nursing homes (registered nurses [RNs], certified nursing assistant [CNAs], nurse aides, direct care staff, and support staff). Minimal variation was noticed in the quality of the studies assessed using CCAT, with scores ranging from 36 (36/40, 90%) to 40 (40/40, 100%) out of a total of 40 (100%) points.

**Table 1:** Characteristics of the included studies

No	Ref. No	Author/year	Country	Sample size	Study design	Population	Tools	CCAT score
1	[11]	Yount, Zebrak, Famolaro, Sorra and Birch, 2020	US	15,726	Quantitative	Providers and staffs	NHSPSC	39 (98%)
2	[12]	Li et al., 2019	US	818	Quantitative	Administrators, directors of nursing, and unit leaders	NHSPSC	37 (93%)
3	[13]	Smith et al., 2018	US	196	Quantitative	Facility staffs	NHSPSC	39 (98%)
4	[15]	Temkin-Greener et al., 2020	US	818	Quantitative	Facility administrators, directors of nursing, and unit nursing leaders	NHSPSC	39 (98%)
5	[16]	Bondevik et al., 2017	Norway	463	Quantitative	Healthcare providers	SAQ-AV	38 (95%)
6	[21]	Bonner, Castle, Men and Handler, 2009	US	1579	Quantitative	CNAs	HSOPSC	40 (100%)
7	[22]	Castle and Sonon, 2006	US	2717	Quantitative	Administrators	HSOPSC	40 (100%)
8	[25]	Laura M Wagner, Brush, Castle, Engberg and Capezut, 2020	US	1133	Quantitative	RNs and licensed practical/vocational nurses	NHSPSC	38 (95%)
9	[26]	Teigne et al., 2019	France	2020	Quantitative	All salaried professionals	NHSPSC	40 (100%)
10	[27]	Castle, Wagner, Perera, Ferguson and Handler, 2010	US	3698	Quantitative	Administrator/manager, licensed nurse, nurse aide, direct care staff, and support staff	NHSPSC	39 (98%)
11	[28]	Castle, Wagner, Perera, Ferguson and Handler, 2009	US	112,319	Quantitative	Staffs	NHSPSC	39 (98%)
12	[29]	Orth, Li, Simning, Zimmerman and Temkin-Greener, 2020	US	11,957	Quantitative	Residents with dementia	NHSPSC	39 (98%)
13	[30]	Seljemo, Viksveen and Ree, 2020	Norway	165	Quantitative	Staff members	NHSPSC	39 (98%)
14	[31]	Ree and Wiig, 2019	Norway	304	Quantitative	Healthcare professionals	HSOPSC	38 (95%)
15	[32]	He et al., 2020	China	549	Quantitative	Staff members	HSOPSC	39 (98%)
16	[33]	Arnetz et al., 2011	US	312	Quantitative	Staff members	HSOPSC	38 (95%)
17	[34]	Castle, 2006	US	1579	Quantitative	Nurse aides	HSOPSC	39 (98%)
18	[35]	Wagner, Capezuti and Rice, 2009	US	551	Quantitative	Licensed nurses	HSOPSC	38 (95%)
19	[36]	Castle et al., 2007	US	2840	Quantitative	Administrators	HSOPSC	39 (98%)
20	[37]	Handler et al., 2016	US	151	Quantitative	Doctors, pharmacists, advanced practitioners, and nurses	HSOPSC	39 (98%)
21	[38]	Wisniewski et al., 2007	US	51	Quantitative	All nursing and allied health care staff	SAQ-SNF	38 (95%)
22	[39]	Deilkas, Hofoss, Husebo and Bondevik, 2019	Norway	288	Quantitative	RNs, nursing assistants, and health workers	SAQ -A	36 (90%)
23	[40]	Gunnar Tschudi Bondevik, Hofoss, Husebø and Deilkås, 2019	Norway	266	Quantitative	RNs and medical doctors	SAQ	38 (95%)
24	[41]	Buljac-Samardzic et al., 2006	Netherlands	521	Quantitative	Employees who provide direct care	SAQ	37 (93%)
25	[42]	Halligan, Zecevic, Kothari, Salmoni and Orchard, 2014	Canada	Focus groups:7, Surveys: 21	Mixed methods	All frontline staff on the unit and management team	MSI	-

*AHRQ* Agency for Healthcare Research and Quality, *CCAT* Crowe Critical Appraisal Tool, *CNA* Certified nursing assistant, *HSOPSC* Hospital Survey on Patient Safety Culture, *MSI* Modified Stanford Patient Safety Culture Survey Instrument, *NH* Nursing home, *NHSPSC* Nursing Home Survey on Patient Safety Culture, *RN* Registered nurse, *SAQ* Safety Attitudes Questionnaire, *SAQ-AV* Ambulatory Version of the Safety Attitudes Questionnaire, *SAQ-SNF* Safety Attitudes Questionnaire in a Skilled Nursing Facility (SNF)

### Comparison of the differences between tools

As shown in Table. 2, all the tools used in the selected studies were analyzed to assess whether the PSC elements—based on the elements included in the HSOPSC version 1.0—were met. The results revealed that there was no “frequency of reported incidents” in the NHSPSC; thus, “procedure compliance” was added. The SAQ tool did not have “communication openness,” “feedback and communication on errors,” “non-punitive response to errors,” “organizational learning,” “employee placement,” “transition and transition,” or “overall awareness of safety;” instead, it measured “job satisfaction,” “working conditions,” and “stress recognition.” The MSI tool consists of seven areas, and compared to the HSOPSC, “Management expectations and actions promoting patient safety” and “Overall perceptions of safety” are included. In addition, more emphasis is placed on safety such as “Perceived State of Safety” and “Senior Leadership Support for Safety.“ Overall, factor 3 of “Teamwork within units,“ factor 8 of “Teamwork across units,“ and factor 10 of “Management support for resident safety” were included in all tools, except for the MSI tool, while factor 6 of “Management expectations and actions promoting patient safety” was included in all tools. On the other hand, factor 11 of “Frequency of events reported” was not included in other tools except for the HSOPSC tool. Its Cronbach’s alpha reliability was reported to range between 0.7 and 0.90.

**Table 2:** Quality assessment of the tools included in the study

Measure	Elements^a^												Score	Measurement properties	
	1	2	3	4	5	6	7	8	9	10	11	12			
HSOPSC_ver 1.0	v	v	v	v	v	v	v	v	v	v	v	v	34item, 5-point Likert scale	Reliability: 0.70 0.81	
NHSPSC	v	v	v	v	v	v	v	v	v	v		v	43item, 5-point Likert scale	Reliability: 0.70 ~ 0.90	
NHSPSC-China	v		v		v	v	v	v	v				23item, 5-point Likert scale	Reliability: 0.884 (0.737 ~ 0.866)	
SAQ			v			v		v		v			30item, 5-point Likert scale	Reliability: 0.90	
SAQ-SNF			v			v		v		v			30item, 5-point Likert scale	Reliability: 0.90	
SAQ-AV			v			v		v		v			62item, 5-point Likert scale	Reliability: 0.886 (0.655 ~ 0.786)	
MSI						v						v	43item, 5-point Likert scale	Reliability: 0.73 ~ 0.89	

*HSOPSC* Hospital Survey on Patient Safety Culture, *NHSPSC* Nursing Home Survey on Patient Safety Culture, *SAQ* Safety Attitudes Questionnaire, *SAQ-SNF* Safety Attitudes Questionnaire in a Skilled Nursing Facility (SNF), *SAQ-AV* Ambulatory Version of the Safety Attitudes Questionnaire, *MSI* Modified Stanford Patient Safety Culture Survey Instrument

^a^Element 1: Communication openness; Element 2: Feedback and communication about error; Element 3: Teamwork within units; Element 4: Non-punitive response to error; Element 5: Organizational learning; Element 6: Supervisor/manager expectations and actions promoting patient safety; Element 7: Staffing; Element 8: Teamwork across units; Element 9: Handoffs and transitions; Element 10: Management support for patient safety; Element 11: Frequency of events reported; Element 12: Overall perceptions of safety; V means the element has been met

### Patient safety culture differences between hospitals and nursing homes

The PSC scores of nursing homes and hospitals differed slightly in terms of their subdomains, although most studies reported that nursing home scores were low [22, 28, 34, 36, 37]. However, one study reported that nursing homes also had higher scores than hospitals in some domains [28]. The PSC score was higher for RNs and CNAs in hospitals with low turnover rates [15]. While each increase in the overall positive response rate to PSC reported a decrease in medical defects (*p* = .001), proven complaints (*p* = .004), and fines (*p* = .059), there was an increase in the probability of being assigned a 4- or 5-star quality rating [12]. However, studies using the NHSPSC reported no sub-factors significantly associated with 5-star ratings [11]. People living with dementia in the nursing home group had a lower risk of in-hospital death, as their openness to communication regarding their PSC scores was higher [29]. The results reveal that higher PSC scores among CNAs are reflected by patients’ falls, for which modulated restraint use was reported [21]. In addition, increasing age and job position were associated with significantly higher mean scores for patient safety factors (teamwork climate, safety climate, job satisfaction, and working conditions) [40].

### Patient safety culture differences between employees

Considering the factors influencing nurses in terms of PSC, the PSC questionnaires reveal that the scores of CNAs were lower than those of RNs [41], while nurse managers reported higher scores than staff nurses [35, 36]. Licensed nurses employed in government-run establishments had significantly lower awareness of a positive safety culture than did nurses employed in nonprofit organizations [35]. RNs, licensed practical nurses, and nurse management/supervisors received the highest ratings for quality of collaboration and communication (very high), whereas nurse practitioners and physician assistants received the lowest ratings (range: 2.5–2.9) [38].

### Factors affecting patient safety

As shown in Table. 3, factors affecting patient safety included transformational leadership, job demands, job resources [30], facility ownership (*p* < .001), facility scale (*p* < .001), reporting management (*p* < .001) [40], being an integrated care institution or not (*p* = .006), frequency of concern about patient safety (*p* = .001), occurrence of adverse events in departments (*p* = .001), and a punitive atmosphere [32]. One study reported a positive correlation between teamwork climate, job satisfaction, perceptions of management, safety climate, and working conditions [37]. In this study, the explanatory power was 42.7%, with staffing and communication openness being significant predictors [31].

**Table 3:** Study aims and main results

No	Ref. No	Study aim	Main result
1	[11]	To identify associations between PSC and health care quality in NH	• None of the NHSPSC measures were significantly associated with the staffing five-star rating.
2	[12]	To determine the associations of PSC with the Nursing Home Compare enforcement outcomes and 5-star ratings on multiple domains of care	Every increase of 10% points in the overall positive response rate for safety culture was associated with • 0.56 fewer healthcare deficiencies (*p* = .001), • 0.74 fewer substantiated complaints (*p* = .004), • Reduced fines by $2285.20 (*p* = .059), • Increased odds of being designated as 4- or 5-star (vs. 1–3 star) facilities (odds ratio roughly 1.20, *p* < .05)
3	[13]	To examine the association between NH safety culture and catheter-associated urinary tract infection rates	• None of the 13 safety culture measures were statistically significant.
4	[15]	To investigate the relationship between employee turnover and PSC in NH	• In NHs with low turnover, the overall PSC scores were 4.04% (RNs) and 6.28% (CNAs), which were higher than scores in NHs with high turnover. • Low turnover of RNs and CNAs exhibited a strong, statistically significant, and positive association with PSC • PSC domains of teamwork, staffing, and training/skills appeared to be particularly related to CNA turnover, but not to RN • PSC domains focusing on collaboration across disciplines and roles—such as compliance with procedures, handoffs, communication openness, and organizational learning—appeared to be equally associated with CNA and RN turnover
5	[16]	(i) To investigate safety attitudes among healthcare providers in NHs, using the SAQ-AV (ii) To investigate whether safety attitudes were related to professional background, age, work experience, and mother tongue	• Increasing age and job position were associated with significantly increased mean scores for patient safety factors (teamwork climate, safety climate, job satisfaction, and working conditions) • Not being a Norwegian native speaker was associated with a significantly higher mean score for job satisfaction and a significantly lower mean score for stress recognition • Neither professional background nor work experience was significantly associated with any patient safety factor.
6	[21]	To examine whether CNAs’ perceptions of PSC were correlated with clinical outcomes (rates of falls, pressure ulcers, and daily restraint use)	• High CNA PSC scores positive association with falls (B = 0.015; *p* = .000) • High CNA PSC scores positive association with moderate restraint use (B = 0.172;* p* = .017) • CNA PSC scores showed no association with pressure ulcer rates.
7	[22]	(i) To determine safety culture scores for NHs (ii) To compare these results with existing data from hospitals	• 11 of the 12 HSOPSC subscale scores from the NH sample were considerably lower than the benchmark hospital scores. •Almost all item scores from NHs were considerably lower than the benchmark hospital scores.
8	[25]	To analyze how nurses’ PSC perceptions corresponded to their personal and professional characteristics	• US-born and educated nurses demonstrated the lowest perceptions of workplace PSC overall (*P* < .001)
9	[26]	To perform a transcultural adaptation in French of the NHSPSC questionnaire	•The NHSPSC questionnaire is the first questionnaire on PSC that has been applied to the medico-social sector in France. • The exploratory analysis led to the identification of seven domains; teamwork, staffing, compliance with procedures, handoffs, feedback and communication about incidents, supervisor expectations and actions promoting resident safety, overall perceptions of resident safety and organizational learning.
10	[27]	To examine the overall responses of NH staff to a newly developed NHSPSC and to examine whether NH staff differ in their PSC ratings	• Staff in NHs generally agree that PSC is poor. • Administrators/managers had more positive scores than did other staff types (*p* < .05) across most domains.
11	[28]	To compare the PSC between nationally representative samples of hospitals and NHs	• Of the 26 highly similar items in these questionnaires, 9 of the NHSPSC scores were lower than the corresponding HSOPSC scores (indicating poorer perceptions of safety culture), 1 score was identical, and 16 were higher (indicating better perceptions of safety culture). • Some learning opportunity may present itself for both nursing homes and hospitals to improve the safety culture.
12	[29]	(i) To examine associations between PSC domains and place of death among residents with dementia (ii) To evaluate the extent to which state minimum NH nurse staffing requirements moderate these effects	• Residents with dementia in NHs with higher PSC scores in communication openness had lower odds of in-hospital death
13	[30]	To assess the association of transformational leadership, job demands, and job resources with PSC and employees’ overall perception of patient safety in NH	• Transformational leadership explained 47.2% of the variance in PSC and 25.4% of the overall perception of patient safety, controlling for age and gender (*p* < .001). • Job demands and job resources explained 7.8% of PSC and 4.7% of the overall perception of patient safety (*p* < .001).
14	[31]	To assess not only staff perceptions of PSC in-home care services and NH, but also how various PSC dimensions contribute to explaining overall perceptions of patient safety	• The number of patient safety dimensions with an average of more than 60% of positive responses was 7 out of 10 in NHs, and 9 out of 10 in-home care. • In-home care, the total explained variance of overall perceptions of patient safety was 45%, with teamwork, staffing, and handoffs being significant predictors. • The explained variance in NHs was 42.7%, with staffing and communication openness being significant predictors
15	[32]	To investigate PSC and its relationship with obstacles to adverse event reporting in Chinese NHs	• PSC in NHs was associated with facility ownership (*p* < .001), facility scale (*p* < .001), reporting management (*p* < .001), being an integrated care institution or not (*p* = .006), frequency of concern about patient safety (*p* = .001), occurrence of adverse events in departments (*p* = .001) and a punitive atmosphere (*p* = .044). Obstacles to adverse event reporting were negatively correlated with PSC (*p* < .05).
16	[33]	To identify organizational climate predictors of specific aspects related to the staff-rated resident safety culture in NHs	• The organizational climate factors “efficiency” and “work climate” predicted non-punitive response to mistakes (p < .001 for both scales) and compliance with procedures (*p* < .05 and *p* < .001, respectively). • Work stress was an inverse predictor of compliance with procedures (*p* < .05). • Goal clarity was the only significant predictor of communication about incidents (*p* < .05).
17	[34]	(i) To compare the resident safety culture of NH from a nurse aide’s perspective with existing data from hospitals (ii) To examine the differences in the safety culture of NHs according to facility and market characteristics	• All of the 12 HSOPSC subscale scores from the NH sample were considerably lower than the benchmark hospital scores, indicating a less well-developed safety culture. • The resident safety culture of nurse aides in many NHs may be poorly developed.
18	[35]	To describe perceptions of workplace safety culture among nurses employed in long-term care settings	• Nurse managers reported significantly more positive safety culture perceptions than did other licensed staff nurses. • Licensed nurses employed in government-run facilities had significantly fewer positive safety culture perceptions than did those working in nonprofit organizations.
19	[36]	(i) To compare the resident safety culture of NHs from a top management perspective with existing data from hospitals. (ii) To examine how the safety culture of NHs varies according to facility and market characteristics	• 9 of the 10 HSOPSC subscale scores from the NH sample were considerably lower than the hospital scores • The resident safety culture reported by administrators was generally low. • High RN staffing is significantly associated with high resident safety scores.
20	[37]	To assess PSC in the NH setting, in order to determine whether NH professionals differ in their PSC ratings, and to compare PSC scores of NHs with those of hospitals	• NHs scored significantly lower than hospitals (*p* = .05) in 5 PSC dimensions (non-punitive response to error, teamwork within units, communication openness, feedback and communication about error, and organizational learning)
21	[38]	To assesses staff attitudes regarding safety culture at one 250-bed SNF	• SAQ is a validated and reliable instrument in the SNF setting • No statistically significant differences were found between nursing and other healthcare staff in ratings of the 6 safety constructs or the quality of collaboration and communication between staff members. • RNs, licensed practical nurses, and nurse management/supervisors received the highest ratings for quality of collaboration and communication (very high), whereas nurse practitioners and physician assistants received the lowest (range: 2.5–2.9).
22	[39]	To investigate whether the Norwegian translation of the SAQ-AV is useful to identify significant variations in the patient safety climate factor scores	• It can be used to identify wards in NHs with high and low risk of adverse events • Staff perceptions of safety climate, working conditions, and perceptions of management varied significantly across wards.
23	[40]	To validate the study using the Norwegian translation of the questionnaire in the primary care setting and present the psychometric properties of this version	• The Norwegian-translated version of the SAQ–AV, with the five confirmed factors, might be a useful tool for measuring several aspects of PSC in primary care settings.
24	[41]	(i) To investigate whether the SAQ is appropriate to measure the safety attitude of caregivers in nursing and residential homes (ii) To compare the safety attitude of these caregivers with available data of caregivers in other settings (i.e., inpatients, intensive care units, and ambulatory care)	• SAQ versions were completely applicable in nursing and residential homes • There were positive correlations between teamwork climate, job satisfaction, perceptions of management, safety climate, and working conditions (r = .31 to 63) • Stress recognition had a negative correlation with each of the other dimensions (r = -.13 to -0.18)
25	[42]	To understand safety culture in a high-risk secured unit for cognitively impaired residents in a long-term care facility	• The respondents perceived the overall state of safety culture to be weak (80% positive response).

*CNA* Certified nursing assistant, *NH* Nursing home, *RN* Registered nurse, *NHSPSC* Nursing Home Survey on Patient Safety Culture, *HSOPSC* Hospital Survey on Patient Safety Culture, *SAQ* Safety Attitudes Questionnaire, *SAQ-SNF* SAQ in a Skilled Nursing Facility (SNF), *SAQ-AV* Ambulatory Version of the Safety Attitudes Questionnaire, *PSC* Patient safety culture

## Discussion

This review aimed to investigate the factors affecting patient safety in older adults living in nursing homes by reviewing previous studies on patient safety in nursing homes. The synthesis of the 25 papers identified revealed that most of the tools used to measure patient safety in nursing homes were related to PSC and employees’ attitudes. In addition, higher PSC scores were found to be associated with lower reported medical defects [8, 11, 12].

PSC was found to be an important factor affecting the safety of nursing home residents. These findings are consistent with existing studies on the effect of PSC on patient safety as an organizational factor [21, 29]. This study is particularly meaningful in that it systematically analyzed the tools developed for nursing homes (NHSPSC), unlike previous studies that reported that the HSOPSC was the only tool to measure PSC in nursing homes in 2008 [21]. The selected literature used the HSOPSC tool developed for hospitals until the AHRQ published the NHSPSC in 2009 to measure PSC in nursing homes [33]. However, even after its publication, although nursing homes were the focus of several studies, HSOPSCs were used for comparison with hospitals [36, 37, 41].

This study revealed that nursing homes generally scored lower than hospitals when using the HSOPSC to measure PSC. The authors attribute this to a dimension related to error reporting. In the case of the US, the federal government is required to report abuses, injuries of an unknown source, mistreatment, among others, of residents in nursing homes [43], but this is not included in the dimension of the NHSPSC tool. According to the results reported by 173 Swedish nursing homes in 2018, 89% of serious adverse reactions occurred due to medication errors, falls, delays or improper interventions, and neglect of care [44]. Therefore, a system for the occurrence of accidents in nursing homes is important, for which the NHSPSC tool may have to be modified in the future.

In addition, the differences in PSC scores between hospitals and nursing homes may reflect the personnel composition and characteristics between the two settings. Unlike hospitals, nursing homes often provide residents with care through nurse assistants or care workers rather than RNs [22]. Many studies have reported that the higher the number of RNs, the better the patient safety and quality indicators [45, 46]. In 2014, 11,339 nursing homes across the US investigated the inappropriate use of antipsychotic drugs in both mentally ill and normal groups of patients [47]. Increasing the number of hours of direct care provided by a nurse assistant instead of by an RN was found to increase the use of psychotropic drugs, sounding a warning about the decrease in the number of RNs. Although the dimension of “procedure compliance” was added to NHSPSC due to the different personnel composition, a program providing better education to those who provide direct care to residents is not only needed but will also be more valuable in ensuring better PSC.

In the US, nursing home evaluations of quality indicators, such as falls, severe pain, pressure sores, urinary tract infections, and physical restraints, have been published on a public website [48]. South Korea also conducts quality assessments of nursing homes, with most studies focusing on structures and processes and failing to include health outcomes for residents [49]. Although information on the overall facility, including the number of RNs, has been disclosed, it does not address the quality and safety of care at the facility. Moreover, in Korean nursing homes with fewer RNs, the number of RNs did not affect the quality of care indicators [50]. With the development of the Korean PSC tool in 2013 [51], interest in patient safety in nursing homes has increased. Therefore, in future research, it is necessary to develop an evaluation system and tool to comprehensively evaluate and measure patient safety in nursing homes, including PSC.

This study has several limitations. First, since this only considered studies that used tools, the results should be cautiously interpreted because studies that employed other designs without the use of tools were excluded. Second, this study compared the tools used in each literature with the version 1.0 domain of the HSOPSC tool. Since version 2.0 has been released, it is necessary to compare them in future studies. Third, since all articles were in English, important articles published in other languages may have been overlooked. Finally, a meta-analysis could not be performed because the literature analyzed in this study was reported using various tools and considering different results. It is necessary for future research to present quantitative evidence for studies that report findings based on using the same tools.

## Conclusion

This study revealed that the PSC of nursing homes is a critical factor that influences the safety of their residents. Therefore, it is necessary to strengthen organizational factors, such as the staff education system and the composition of appropriate personnel, for establishing and fostering a PSC in nursing homes, for which policy support is also essential.

## Data Availability

All data generated or analyzed during this study are included in this published article.

## Abbreviations

AHRQ: Agency for Healthcare Research and Quality
CCAT: Crowe Critical Appraisal Tool
CNAs: certified nursing assistants
HSOPSC: Hospital Survey on Patient Safety Culture
MSI: Modified Stanford Patient Safety Culture Survey Instrument
NH: nursing home
NHSPSC: Nursing Home Survey on Patient Safety Culture
PSC: Patient Safety Culture
RNs: registered nurses
SAQ: Safety Attitudes Questionnaire
SAQ-AV: Ambulatory Version of the Safety Attitudes Questionnaire
SAQ-SNF: Safety Attitudes Questionnaire in a Skilled Nursing Facility
